# The effects of a 5-year physical activity on prescription (PAP) intervention in patients with metabolic risk factors

**DOI:** 10.1371/journal.pone.0276868

**Published:** 2022-10-31

**Authors:** Stefan Lundqvist, Åsa Cider, Maria E. H. Larsson, Lars Hagberg, Marcus Praetorius Björk, Mats Börjesson

**Affiliations:** 1 Department of Health and Rehabilitation, Unit of Physiotherapy, Institute of Neuroscience and Physiology, Sahlgrenska Academy, University of Gothenburg, Gothenburg, Sweden; 2 Center for Physical Activity Gothenburg, Regionhälsan, Region of Västra Götaland, Gothenburg, Sweden; 3 Department of Occupational Therapy and Physiotherapy, Sahlgrenska University Hospital, Gothenburg, Sweden; 4 Region Västra Götaland, Research, Education, Development and Innovation, Primary Health Care, Gothenburg, Sweden; 5 Faculty of Medicine and Health, University Health Care Research Center, Örebro University, Örebro, Sweden; 6 General Practice/ Family Medicine, School of Public Health and Community Medicine, Institute of Medicine, Sahlgrenska Academy, University of Gothenburg, Gothenburg, Sweden; 7 Center for Health and Performance (CHP), University of Gothenburg, Gothenburg, Sweden; 8 Department of Acute and Molecular Medicine, Institute of Medicine, Sahlgrenska Academy, University of Gothenburg, Gothenburg, Sweden; 9 Department of MGAÖ, Sahlgrenska University Hospital, Region of Västra Götaland, Gothenburg, Sweden; University of South Australia, AUSTRALIA

## Abstract

**Background:**

Increased physical activity (PA) has positive effects on health and longevity. In Swedish healthcare, the physical activity on prescription (PAP) method reportedly increases patients’ PA levels for up to 12 months, but long-term follow ups are lacking. As it remains difficult to maintain lifestyle changes, our aim was to evaluate adherence and clinical effects at a 5-year follow-up of PAP treatment in primary healthcare.

**Methods:**

This longitudinal, prospective cohort study included 444 patients, (56% female), aged 27–85 years, with at least one metabolic risk factor. Participants were offered PAP by nurses or physiotherapists. The PAP intervention included an individualised dialogue, a PA recommendation by written prescription, and individually adjusted follow-up over 5 years, according to the Swedish PAP model. Patient PA level, metabolic risk factors, and health related quality of life (HRQoL) were measured at baseline and at the 6-month, 1.5-year, 2.5-year, 3.5-year, and 5-year follow-ups. Estimated latent growth curves were used to examine levels and rates of change in the outcomes.

**Results:**

The study dropout rate was 52%, with 215 of 444 patients completing the 5-year follow-up. At follow-up, the mean PA level had increased by 730 MET-minutes per week or 3 hours of moderate-intensity PA/week when compared to baseline. During the 5-year intervention, we observed significant positive changes (p ≤ 0.05) in 9 of 11 metabolic risk factors and HRQoL parameters: body mass index, waist circumference, systolic and diastolic blood pressure, fasting plasma glucose, triglycerides, cholesterol, high-density lipoprotein, and mental component summary.

**Conclusion:**

This first evaluation of a 5-year PAP intervention in primary care demonstrated positive long-term (5 years) effects regarding PA level, metabolic health, and HRQoL. The recorded long-term adherence was ~50%, which is in line with medical treatment. Despite limitations, PAP can have long-term effects in an ordinary primary care setting.

## Introduction

Firmly established evidence supports the positive effects of physical activity (PA) on health and longevity [1,2] and regular PA is considered essential for the prevention and treatment of several diseases [3]. Metabolic syndrome (MetS), which includes being overweight, and exhibiting abdominal obesity, insulin resistance, dyslipidaemia, and hypertension in various combinations, increases the risks of cardiovascular disease (CVD), type 2 diabetes (DM), and premature death [4,5], and is positively influenced by regular PA [6,7]. The recently updated recommendations for physical activity among adults include a target of 150–300 min per week of moderate-intensity aerobic PA, or 75–150 min per week of vigorous-intensity aerobic PA [2]. Notably, PA of any duration, even below the recommended threshold, is important for health and being somewhat active is better than doing nothing. Indeed, there is a dose-response relationship between PA and MetS prevalence, with low-intensity PA being associated with a 50% lower MetS prevalence, and moderate- to vigorous-intensity PA with a 67% lower MetS prevalence, compared to no activity [8].

Long-term follow-up of >7 years shows that both metabolically “healthy” (i.e., without any known additional cardiovascular risk factors) obese individuals and metabolically unhealthy (having risk factors) normal weight individuals, carry increased risks of developing CVD and DM [9]. Importantly, positive changes in risk factors included in MetS can yield reduced risks of CVD and all-cause mortality over 7–9 years [10]. The positive metabolic effects of increased PA highlight the importance of identifying interventions in regular healthcare that may have positive long-term effects on increased PA levels in patients.

Several methods to increase patients’ PA levels have been introduced in healthcare, with varying individualization and success [11–14]. In Swedish healthcare, the physical activity on prescription (PAP) method, which has been used during the last 20 years to help patients increase their PA levels [15], includes a patient-centred dialogue; an individually tailored PA recommendation, including a written prescription; and an individualised follow-up. All licensed healthcare professionals can offer PAP treatment for preventive and therapeutic purposes, or as a complementary or first-line treatment [16,17].

A systematic review of Swedish PAP treatment reveals that it effectively increased PA levels among insufficiently active patients [18], for at least 12 months. From the patient’s perspective, tailored PAP including a written prescription and regular follow-up is considered important for increasing and maintaining motivation and PA level [19–22], and may contribute to the efficacy of the PAP method. Long-term adherence is essential, and a proxy of behavioural change. In a recent systematic review and meta-analysis, Arsenijevic et al. indicate that the type and duration of intervention programme may influence both the adherence rate and efficacy [23]. However, earlier clinical studies of lifestyle behavioural change show low levels of long-term adherence [24], while PAP has shown short-term adherence at a similar level to medical care [25,26]. Thus, further studies are needed to evaluate Swedish PAP treatment from a long-term perspective, and for different patient subgroups [23,27]. PAP is still insufficiently implemented in Swedish healthcare [28,29].

In the present part of the *Gothenburg PAP study*, we aimed to evaluate a 5-year period of PAP treatment in primary healthcare for adult patients who were physically inactive and had metabolic risk factors. The goal was to explore possible long-term changes in PA level, metabolic health, and health-related quality of life (HRQoL), as well as adherence to the PAP intervention over time.

## Methods

### Study design

A longitudinal, prospective, cohort study of PAP treatment over 5 years was carried out, mainly in the primary care setting at daily clinical healthcare centres (HCCs). The study design has been previously described in detail [30–32]. The study was conducted in accordance with the ethical principles described in the Declaration of Helsinki and was approved by the Regional Ethical Review Board in Gothenburg, Sweden (Dnr 529–09 and Dnr 678–14).

### Study population

**Fig 1:**
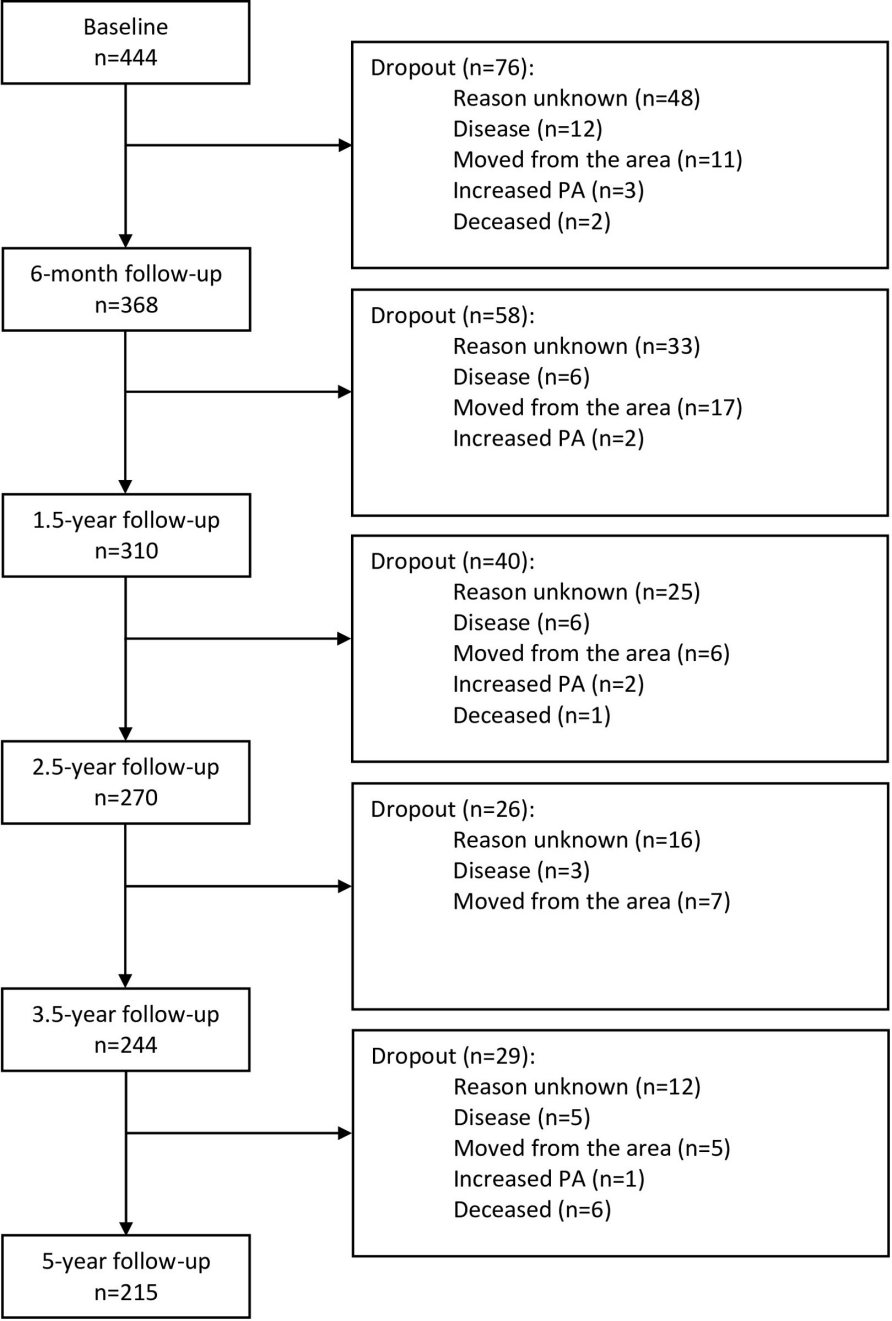
Flow of patients involved in the study. The patients were recruited from 15 health care centres.

Between 2010–2014, this study included 444 patients, 56% of whom were women, aged 27–85 years (mean age 57 years), with metabolic risk factors and insufficient physical activity (<150 min/week). The patients were recruited from 15 primary healthcare centres in Gothenburg as a convenience sample, and all agreed, both orally and in writing, to participate in the PAP treatment. The 6-month follow-up was completed by 368 patients (dropout rate: 17%), 156 of whom had achieved a sufficient PA level of 150 min/week and continued ordinary PAP treatment at the HCC (>150 HCC group). At this time, the 190 patients reporting a PA level < 150 min/week were randomized to receive either continued ordinary PAP treatment at the HCC (<150 HCC group, n = 92) or PAP treatment supported by a physiotherapist (PT) (<150 PT group, n = 98). The remaining 22 patients in the study either declined the offered randomisation or lacked PA data, and continued ordinary PAP treatment at the HCC, and thus were not included in the current analysis (Fig 1).

### Intervention

At the HCC, PAP treatment was offered by nurses educated on the health effects of PA and on PAP treatment. The intervention included an individualised dialogue concerning PA, an individually dosed PA recommendation with a written prescription, and an individually adjusted follow-up [30], following the concept of the Swedish PAP model [16] and the *Physical Activity in the Prevention and Treatment of Disease* (FYSS) reference handbook [15].

The PAP intervention offered by PTs, who were also educated in PAP treatment, included the individualised dialogue and the individual PA recommendation [31]. However, the third part (follow-up) was arranged via a fixed follow-up schedule comprising a total of eleven follow-up sessions during the intervention period: six during the first year of intervention, three during the second year, and one each at the 3-year, and 4.5-year follow-ups, respectively. Additionally, the PT group received six aerobic physical fitness tests using an ergometer bicycle during the intervention period. The results from these tests formed the basis for a continuing dialogue with the patient concerning the choice and individual dosage of PA, which was recorded in a written prescription.

### Measurements

All measurements, except the physical fitness test, were performed at the sampling unit and by the ordinary nurses at the HCCs at baseline and at the 6-month, 1.5-year, 2.5-year, 3.5-year, and 5-year follow-ups.

#### PA level

PA was assessed using two questionnaires. First, the American College of Sports Medicine (ACSM)/American Heart Association (AHA) questionnaire was used to assess whether the patient had reached the recommended PA level of 150 min/week. Patients received 1 point if they were physically active at a moderate intensity level for 30 min per day, and 1.7 points if they were physically active at a more vigorous intensity level for 20 min per day. A weekly score of <5 points indicated an inadequate PA level, according to public health recommendations of the ACSM and AHA [33]. Secondly, the International Physical Activity Questionnaire (IPAQ) was used to score the reported duration (min) and frequency (days) of three specific types of PA performed during the past 7 days: walking, moderate-intensity activities, and vigorous-intensity activities. The results are presented as either a score from three categories—low, moderate, and high PA level—or as energy expenditure estimated as median metabolic equivalent (MET)-minutes per week, with a total MET-minutes/week (TotalMET) summarized from the three types of PA: walking, moderate-intensity activity, and vigorous-intensity activity. A summary score of <600 MET-minutes/week was considered an inadequate (low) PA level [34,35] according to the previously mentioned public health recommendations [33].

#### Anthropometrics

To determine BMI (kg/m2), body weight was measured with the patient wearing light clothing and no shoes, estimated to the nearest 0.1 kg (electric scale; Carl Lidén AFW D300, Jönköping, Sweden). Body height was measured with the patient in an upright position, without shoes, estimated to the nearest 0.5 cm (scale fixed to the wall; PEM 136, Hultafors, Sweden). Waist circumference (WC) was measured with the patient standing, after exhaling air from the lungs. A measuring tape (Kirchner Wilhelm, Aspberg, Germany) was placed on the patient´s skin, between the lower rib and the iliac crest, and the WC was estimated to the nearest 0.5 cm.

#### Blood pressure

Systolic and diastolic blood pressure (SBP, DBP) were measured (in mmHg) with the patient seated, after 5 min of rest [36]. The blood pressure sphygmomanometer (Omron HEM-907, Kyoto, Japan) was attached to the right upper arm at the level of the heart.

#### Blood samples

Blood samples were analysed to determine the levels of fasting plasma glucose (FPG) after an overnight fast, triglycerides (TG), total cholesterol (Chol), high-density lipoprotein (HDL), and low-density lipoprotein (LDL), all expressed in mmol/L. The blood samples were analysed according to the European Accreditation system [37].

#### The cut-off values of MetS components

Cut-off values for MetS components were selected based on the National Cholesterol Education Program (NCEP) classification—WC: >88 cm for women, >102 cm for men; BP: ≥130/85 mm Hg; FPG: ≥6.1 mmol/L; TG: ≥1.7 mmol/L; and HDL: <1.3 mmol/L for women, <1.0 mmol/L for men [38].

#### Health-related quality of life

The Swedish version of the Short Form 36 (SF-36 Standard Swedish Version 1.0) was used to measure HRQoL [39]. The 36 questions covered eight health concepts, which were grouped to express the physical component summary (PCS) and the mental component summary (MCS). These scores were converted to a range of 0–100 points, where higher values represented a better HRQoL.

### Statistical analysis

Baseline data were presented as the mean (standard deviation; SD), median (minimum-maximum; min-max), or number (percentage; %). Baseline differences between the follow-up group vs. dropout group, and between the >150 HCC group vs. <150 PT/HCC group were analysed using the independent samples t-test or Mann-Whitney U-test, based on the data level and the data distribution. For the whole group, characteristics regarding physical activity level, anthropometrics, metabolic risk factors, and health-related quality of life were presented as mean (SD) at each measurement time-point.

According to the 5-year data analysis, we estimated individual differences in the levels and rates of linear and quadratic changes in measurements of health, using latent growth curve (LGC) models with repeated measures (i.e., time) nested within individuals. Models were estimated with structural equation modelling techniques in RStudio version 1.4.1106, using the latent variable analysis (lavaan) package [40]; for more information about LGC models, see [41,42]. All models used full information maximum likelihood (FIML) estimation [43], which is robust against a missing at random missing data assumption. The FIML procedure uses all available information to compute parameters (i.e., both partially complete and fully complete cases are used in the estimation), such that cases with partially missing data on the study variables can still be used in the analysis. The time factors were specified as 1-year linear effects over the study period. Due to non-normal data distribution, TG, FPG, and TotalMet were log-transformed.

## Results

### Study population

Of the 444 included patients, 215 completed the 5-year follow-up, amounting to a 52% dropout rate (Fig 1).

### Baseline characteristics

The baseline characteristics of the whole group have been previously described (30). In summary, the participants’ mean age was 57 years, 56% were female, and a majority had at least two components of MetS (72% WC and BP, 53%WC and TG). A majority (61%) were taking medications for metabolic risk factors, including 54% for arterial hypertension (Table 1). A majority of the patients estimated that they had a low PA level, corresponding to an average of 1–2 brisk 30-minute walks per week (Table 2).

**Table 1:** Baseline characteristics of the follow-up and dropout group.

Variable	Follow-up (n = 215)	Dropout (n = 229)	*p* value
**Age**^a^–years	56.9 (10.6)	58.0 (11.9)	0.328^c^
**Sex** ^b^			0.498^d^
Female	118 (54.9)	133 (58.1)	
Male	97 (45.1)	96 (41.9)	
**Social situation** ^b^			**0.001** ^d^
Single	63 (30.6)	107 (48.2)	
Married/cohabit	133 (64.6)	105 (47.3)	
Other	10 (4.9)	10 (4.5)	
**Economic status**^b^–perceived			0.063^d^
Good	134 (63.8)	115 (52.3)	
Neither nor	59 (28.1)	67 (30.5)	
Bad	17 (8.1)	38 (17.3)	
**Education** ^b^			0.060^d^
Elementary grade	35 (16.7)	48 (21.6)	
Upper secondary school	78 (37.1)	89 (40.1)	
University college	97 (46.2)	85 (38.3)	
**Tobacco** ^b^			0.857^d^
Smokers	14 (6.7)	30 (11.6)	
Non-smokers	144 (68.6)	126 (62.1)	
Ex-smokers	52 (24.8)	65 (26.3)	
**Part of metabolic syndrome** ^b^			
Overweight/Obesity	196 (91.2)	208 (91.2)	0.981^d^
Hyperglycaemia	76 (35.8)	98 (43.9)	0.085^d^
Hypertension	171 (79.5)	175 (77.4)	0.592^d^
Hyperlipidaemia	124 (57.9)	129 (57.1)	0.855^d^
Other diagnosis			
Mental health, depression	26 (12.2)	39 (17.5)	0.122^d^
Musculoskeletal disorders	28 (13.1)	49 (22.0)	**0.016** ^d^
Other	84 (39.4)	109 (48.7)	0.053^d^
**Drug treatment** ^b^			
Overweight/Obesity	1 (0.5)	1 (0.4)	0.974^d^
Hyperglycemia	20 (9.4)	39 (17.5)	**0.014** ^d^
Hypertension	115 (54.0)	121 (54.3)	0.955^d^
Hyperlipidemia	39 (18.3)	55 (24.7)	0.107^d^
Other drug treatment			
Mental health, depression	26 (12.2)	38 (17.0)	0.154^d^
Musculoskeletal disorders	24 (11.3)	36 (16.1)	0.140^d^
Other	70 (32.9)	97 (43.5)	**0.023** ^d^

PT, physiotherapist; HCC, health care centre.

Data are given as ^a^ mean (standard deviation), as ^b^ number (percentage).

Difference between follow-up group and dropout group. *P*-value was determined by ^c^ an independent samples t-test or by ^d^ a Mann-Whitney U-test. Statistical significance was set at *p* ≤ 0.05.

**Table 2:** Baseline characteristics in physical activity, anthropometrics, metabolic risk factors, and health related quality of life for the follow-up and dropout group.

Variable	Follow-up (n = 215)	Dropout (n = 229)	*p* value
Physical activity level			
ACSM/AHA questionnaire^a^, score	1.8 (1.5)	1.6 (1.5)	0.158^d^
IPAQ 1-3^b^, score	1 (1–2)	1 (1–2)	0.702^e^
IPAQ 1-3^c^, category			
• Low	108 (61.4)	114 (63.3)	
• Moderate	68 (38.6)	66 (36.7)	
• High	0	0	
BMI^a^, kg/m^2^	31.7 (5.0)	32.6 (5.5)	0.065^d^
Waist circumference^a^, cm	107.0 (13.1)	109.1 (13.2)	0.109^d^
Blood pressure^a^, mm/Hg			
Systolic	137.6 (17.5)	136.5 (17.9)	0.487^d^
Diastolic	83.4 (11.0)	81.0 (9.2)	0.016^d^
Metabolic components^a^, mmol/l			
Fasting plasma glucose	6.1 (1.5)	6.4 (2.2)	0.086^d^
Triglycerides	1.7 (1.1)	1.7 (0.9)	0.588^d^
Cholesterol	5.5 (1.1)	5.6 (1.3)	0.398^d^
HDL	1.4 (0.4)	1.4 (0.5)	0.537^d^
LDL	3.6 (1.0)	3.7 (1.1)	0.481^d^
HRQOL SF-36^a^, score			
Physical component summary	46.5 (9.4)	43.4 (10.6)	0.002^d^
Mental component summary	43.9 (13.6)	43.5 (13.4)	0.726^d^

PT, physiotherapist; HCC, health care centre; ACSM, American College of Sports Medicine; AHA, American Heart Association; IPAQ, International Physical Activity Questionnaire; MET, metabolic equivalent; BMI, body mass index; HDL, high density lipoprotein; LDL, low density lipoprotein; HRQOL SF-36, health related quality of life 36-Item Short Form Health Survey.

Data are given as ^a^ mean (standard deviation), as ^b^ median (min-max), or as ^c^ number (percentage).

Difference between follow-up group and dropout group. *P*-value was determined by ^d^ an independent samples t-test or by ^e^ a Mann-Whitney U-test. Statistical significance was set at *p* ≤ 0.05.

In the dropout group, participants were more commonly single, and higher proportions had musculoskeletal disorders and received drug treatment for hyperglycaemia and other diagnoses (e.g., respiratory, neurological, rheumatological, and endocrine diseases) (Table 1). Compared to participants who attended the 5-year follow-up, those in the dropout group also had a lower DBP, and lower quality of life PCS-score at baseline (Table 2).

We also performed a subgroup analysis of baseline values between the non-randomised group (>150 HCC) and the randomised groups (<150 PT and <150 HCC). The results revealed few differences, except that the >150 HCC group had a higher PA level at baseline, and lower BMI and WC, as shown in the (S1 and S2 Tables).

### Outcomes: 5-year intervention

#### Whole group analysis

**Fig 2:**
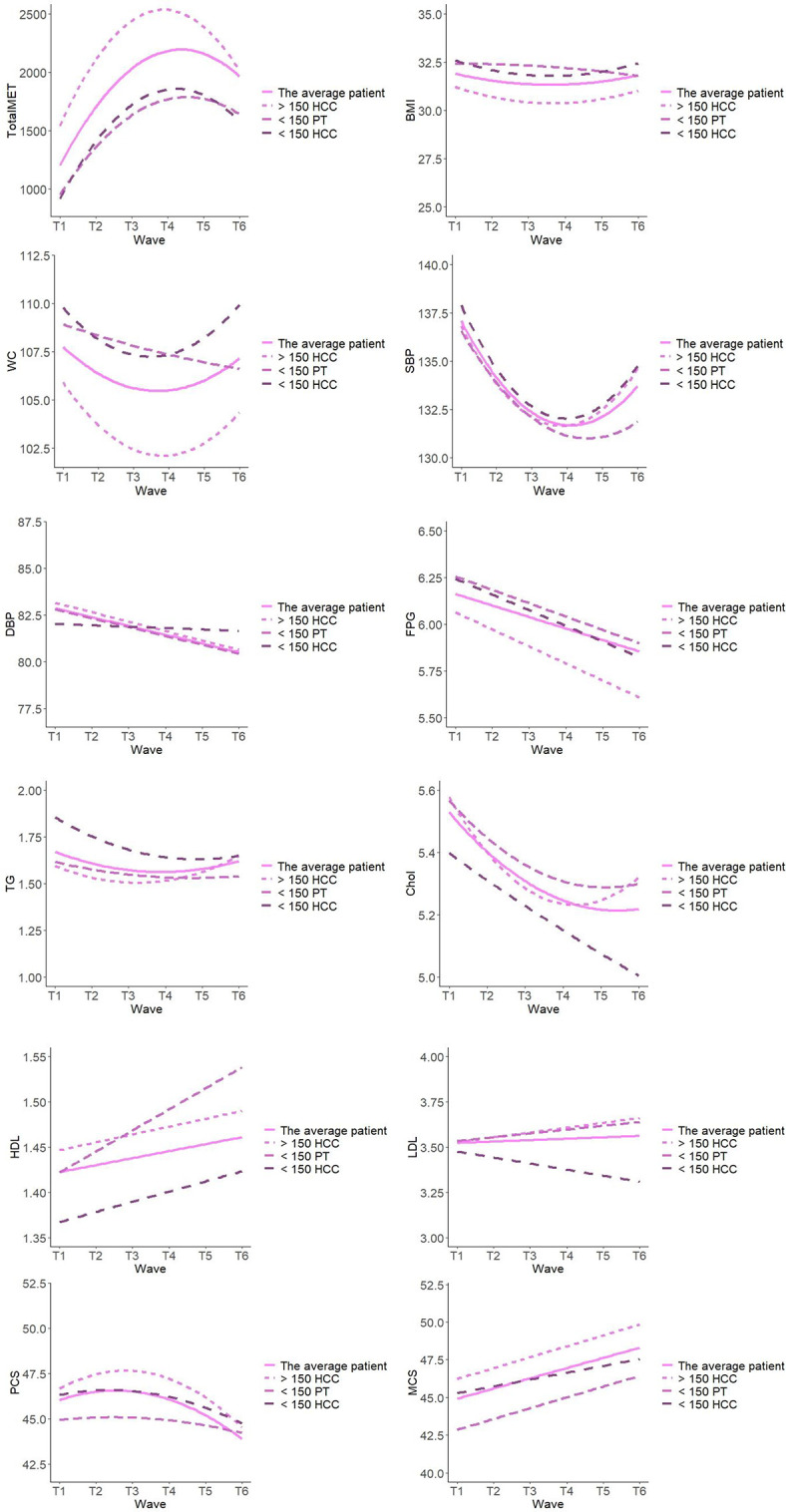
Physical activity level and health outcomes over time for the whole group, and >150 HCC, <150 PT, and <150 HCC subgroups respectively ^a^. MET, metabolic equivalent (minutes/week); BMI, body mass index (kg/m^2^); WC, waist circumference (cm); SBP, blood pressure (mm/Hg); DBP, diastolic blood pressure (mm/Hg); FPG, fasting plasma glucose (mmol/L); TG, triglycerides (mmol/L); Chol, cholesterol (mmol/L); HDL, high-density lipoprotein (mmol/L); LDL, low-density lipoprotein (mmol/L); PCS, physical component summary (score); MCS mental component summary (score). ^a^ Analysed with univariate latent growth curve models separately for each of the measurement.

The results revealed a statistically significant positive change, when analysing the whole study group, over the study period in ten of the twelve measured outcomes: TotalMET, BMI, WC, SBP, DBP, FPG, TG, Chol, HDL, and MCS (Tables 3 and 4). BMI, WC, SBP, TG, and Chol showed similar patterns of change, in which the levels significantly decreased over the study period; these decreases waned over time (Fig 2), but did not return to baseline values. DBP and FPG both exhibited significant linear declines over the study period. HDL and TotalMET showed linear increases, which also waned over time. The LDL and MCS levels significantly increased over the study period, while PCS levels decreased over the study period, a trend that accelerated over the course of the study.

**Table 3:** Characteristics in physical activity level, anthropometrics, metabolic risk factors and health related quality of life, for the whole study group, at each time of measurement ^a^.

		T1–Baseline		T2–6-month		T3–1.5-year		T4–2.5-year		T5–3.5-year		T6–5-year
	n	M (SD)	n	M (SD)	n	M (SD)	n	M (SD)	n	M (SD)	n	M (SD)
TotalMET***	287	1121.6 (1348.3)	264	1794.6 (1962.8)	226	2190.2 (2302.3)	191	1851.6 (1879.4)	188	2028.9 (2242.4)	159	1898.4 (1810.7)
BMI***	341	31.9 (5.2)	338	31.6 (5.3)	285	31.2 (5.3)	250	31.3 (5.2)	226	31.5 (5.4)	199	31.5 (5.6)
WC***	339	107.9 (13.4)	342	106.1 (14.0)	286	104.7 (14.0)	253	105.3 (13.4)	227	105.8 (13.4)	195	106.1 (14.1)
SBP***	343	137.3 (17.7)	340	133.8 (16.3)	288	131.6 (15.4)	254	131.8 (15.9)	228	133.5 (16.1)	202	133.0 (15.5)
DBP**	342	83.0 (10.2)	340	82.6 (9.2)	288	81.2 (9.3)	254	81.0 (9.3)	228	81.8 (9.0)	201	81.1 (8.7)
FPG**	341	6.2 (1.9)	340	6.0 (1.5)	284	6.1 (1.8)	248	6.0 (1.7)	224	5.4 (1.6)	199	6.0 (1.5)
TG*	343	1.7 (1.0)	340	1.6 (0.9)	287	1.5 (0.9)	252	1.6 (1.1)	227	1.6 (1.2)	198	1.6 (0.8)
Chol**	344	5.5 (1.2)	341	5.4 (1.2)	287	5.3 (1.1)	253	5.2 (1.1)	227	5.3 (1.3)	199	6.0 (1.5)
HDL***	345	1.4 (0.4)	341	1.4 (0.6)	287	1.5 (0.5)	252	1.5 (0.5)	227	1.5 (0.5)	199	1.5 (0.4)
LDL	340	3.6 (1.1)	339	3.5 (1.0)	286	3.4 (1.0)	252	3.4 (1.0)	226	3.9 (1.5)	200	3.4 (1.1)
PCS	328	45.8 (9.9)	315	47.0 (9.8)	252	46.4 (10.2)	230	45.7 (10.7)	208	46.1 (10.1)	189	44.4 (11.0)
MCS**	328	44.5 (13.2)	315	46.3 (11.7)	252	46.8 (11.7)	230	46.3 (12.3)	208	47.5 (12.0)	189	48.3 (11.4)

T, time; M, mean; SD, standard deviation; MET, metabolic equivalent (minutes/week); BMI, body mass index (kg/m^2^); WC, waist circumference (cm); SBP, systolic blood pressure (mm/Hg); DBP, diastolic blood pressure (mm/Hg); FPG, fasting plasma glucose (mmol/L); TG, triglycerides (mmol/L); Chol, cholesterol (mmol/L); HDL, high-density lipoprotein (mmol/L); LDL, low-density lipoprotein (mmol/L); PCS, physical component summary (score); MCS, mental component summary (score).

^a^ Data are given as mean (standard deviation).

Statistically significant positive change over the study period in ten of the twelve measured outcomes was marked in the table as: *p < .05, ** p < .01, *** p < .001.

**Table 4:** Changes in physical activity level, anthropometric-, metabolic characteristics and health related quality of life over the study period of 5 years ^a^.

A						
	TotalMET	BMI	WC	SBP	DBP	FPG
	β (S.E)	β (S.E)	β (S.E)	β (S.E)	β (S.E)	β (S.E)
Fixed Effects						
Level	6.93*** (0.08)	31.20*** (0.42)	105.65*** (1.07)	136.74*** (1.36)	83.26*** (0.71)	1.78*** (0.02)
Group (> 150 HCC ref.)						
< 150 PT	-0.45*** (0.13)	1.25 (0.67)	3.48* (1.72)	-0.06 (2.10)	-0.31 (1.14)	0.02 (0.03)
< 150 HCC	-0.46*** (0.13)	1.31 (0.68)	4.14* (1.76)	1.34 (2.23)	-1.23 (1.16)	0.03 (0.03)
Rate of change	0.39*** (0.07)	-0.59*** (0.13)	-2.01*** (0.38)	-3.35*** (0.92)	-0.52** (0.18)	-0.02** (0.01)
Group (> 150 HCC ref.)						
< 150 PT	-0.09 (0.12)	0.31 (0.21)	0.81 (0.60)	0.06 (1.48)	-0.17 (0.29)	0.01 (0.01)
< 150 HCC	-0.07 (0.02)	0.16 (0.21)	0.25 (0.62)	-0.66 (1.51)	0.39 (0.30)	0.01 (0.01)
Change in rate of change	-0.07*** (0.02)	0.12*** (0.03)	0.38*** (0.08)	0.60*** (0.18)	-	-
Group (> 150 HCC ref.)						
< 150 PT	0.03 (0.02)	-0.09* (0.05)	-0.22 (0.13)	-0.17 (0.29)	-	-
< 150 HCC	0.02 (0.02)	-0.04 (0.05)	-0.01 (0.14)	0.08 (0.30)	-	-
Random Effects						
Level	0.26** (0.08)	25.44*** (2.05)	166.14*** (13.69)	188.07*** (20.63)	50.72*** (6.00)	0.04*** (0.01)
Rate of change	0.06 (0.07)	0.91*** (0.21)	7.23*** (1.76)	23.71* (10.91)	1.08** (0.37)	0.01* (0.01)
Change in rate of change	0.01 (0.01)	0.06*** (0.05)	0.35*** (0.08)	0.67 (0.41)	-	-
Residuals	0.62*** (0.04)	1.68*** (0.09)	14.60*** (0.77)	118.06*** (5.99)	47.08*** (2.06)	0.03*** (0.01)
B						
	TG	Chol	HDL	LDL	PCS	MCS
	β (S.E)	β (S.E)	β (S.E)	β (S.E)	β (S.E)	β (S.E)
Fixed Effects						
Level	0.34*** (0.04)	5.57*** (0.10)	1.42*** (0.04)	3.59*** (0.09)	46.75*** (0.80)	46.17*** (0.96)
Group (> 150 HCC ref.)						
< 150 PT	0.03 (0.06)	-0.01 (0.16)	-0.02 (0.06)	0.02 (0.13)	-2.14 (1.27)	-3.29* (1.53)
< 150 HCC	0.15* (0.06)	-0.13 (0.16)	-0.09 (0.06)	-0.09 (0.14)	-0.45 (1.30)	-1.16 (1.56)
Rate of change	-0.05* (0.02)	-0.17** (0.06)	0.05*** (0.02)	0.02 (0.02)	0.84 (0.52)	0.80** (0.22)
Group (> 150 HCC ref.)						
< 150 PT	0.02 (0.03)	0.05 (0.09)	0.01 (0.03)	-0.03 (0.04)	0.07 (0.82)	-0.21 (0.35)
< 150 HCC	-0.01 (0.03)	0.04 (0.09)	0.01 (0.03)	-0.03 (0.04)	-0.62 (0.85)	-0.21 (0.36)
Change in rate of change	0.01* (0.01)	0.02* (0.01)	-0.01** (0.01)	-	-0.30** (0.11)	-
Group (> 150 HCC ref.)						
< 150 PT	-0.01 (0.01)	-0.01 (0.02)	0.01 (0.01)	-	0.11 (0.17)	-
< 150 HCC	-0.01 (0.01)	-0.01 (0.02)	-0.01 (0.01)	-	0.17 (0.17)	-
Random Effects						
Level	0.17*** (0.02)	1.09*** (0.12)	0.16*** (0.01)	0.69*** (0.09)	64.54*** (7.60)	104.17*** (10.79)
Rate of change	0.01 (0.01)	0.09* (0.04)	0.01*** (0.01)	0.02** (0.01)	4.62 (3.41)	1.76*** (0.52)
Change in rate of change	0.01 (0.01)	0.01 (0.01)	-	-	0.18 (0.13)	-
Residuals	0.07*** (0.01)	0.45*** (0.02)	0.04*** (0.01)	0.63*** (0.03)	35.89*** (1.96)	58.22*** (2.69)

MET, metabolic equivalent (minutes/week); BMI, body mass index (kg/m^2^); WC, waist circumference (cm); SBP, systolic blood pressure (mm/Hg); DBP, diastolic blood pressure (mm/Hg); FPG, fasting plasma glucose (mmol/L); TG, triglycerides (mmol/L); Chol, cholesterol (mmol/L); HDL, high-density lipoprotein (mmol/L); LDL, low-density lipoprotein (mmol/L); PCS, physical component summary (score); MCS, mental component summary (score).

^a^ Univariate latent growth curve models were used as statistical method, separately for each of the measurement. Statistical significance was set at *p* ≤ .05: *p < .05, ** *p* < .01, *** p < .001.

Table 3 presents the participants’ characteristics regarding physical activity level, anthropometrics, metabolic risk factors, and health-related quality of life at each follow-up time-point, for the whole study group. Table 4 presents the changes in physical activity level, anthropometric-, metabolic characteristics and health related quality of life over the study period of 5 years for the whole group and >150 HCC, <150 PT, and <150 HCC subgroups respectively. To illustrate the changes, Fig 2 shows these characteristics for the whole group and for the three subgroups respectively.

#### Subgroup analysis

We identified few differences among the three subgroups (>150 HCC, <150 PT, and <150 HCC), with the groups showing similar overall patterns of change. Notably, compared to the <150 PT group, the >150 HCC group had significantly lower WC levels, and higher levels of TotalMET and MCS (Table 4). Additionally, compared to the <150 HCC, the >150 HCC group exhibited significantly lower levels of WC and TG, and higher levels of TotalMET. The groups did not significantly differ in the rates of change over the study period, although it was an overall trend that the <150 PT group showed stronger increases in the level of health in several outcomes (Fig 2).

Adjustment for confounders, sex, age, civil status, economy, education, and smoking revealed few and small differences, see (S1 File). Compared to men, women had significantly lower levels of WC, TG, and FPG, and significantly higher levels of Chol and HDL. Higher age was associated with lower BMI and PCS, and with higher levels of SBP, HDL, and MCS. Gender and age had no effect on the rates of change.

Compared to participants who were in a relationship, those who were single had higher levels of FPG, but showed a greater decline during the study period. Compared to participants who had never smoked, those who had smoked but quit showed higher levels of BMI, WC, and FPG, and lower SBP. Those who smoked at the study baseline showed a steeper decline in TG, while smoking was related to a steeper decline but opposite quadratic effect in Chol (S1 File).

## Discussion

The main findings of the present study were that PAP treatment was associated with long-term adherence and positive long-term effects on PA level, metabolic health, and HRQoL, at the 5-year follow-up in patients with metabolic risk factors.

Of the twelve tested outcome measures, ten showed a statistically significant positive change during the 5-year intervention, with the maximal effect seen after 3–4 years. The degree of improvement of PA was 730 MET-minutes per week (median value), corresponding to approximately 3 hours of moderate-intensity PA per week. This amount of PA is highly clinically important, in light of the global recommendations of 150–300 minutes/week (2.5–5 hours/week) of moderate-intensity PA. There is a known dose-response association between PA and outcome parameters, such as cardiovascular mortality and all-cause mortality [44,45]. A previous study showed that even light-intensity PA for 3 hours per week was associated with a 40% reduction of mortality risk in elderly men [44]. In another study, 15 min per day of moderate intensity PA was associated with a 14% reduced risk of all-cause mortality, and physically active adult men and women showed a 3-year longer life expectancy compared to inactive individuals [45]. The increased PA level in our present study corresponds to, or is greater than, the levels of change achieved in these previous studies, suggesting that the presently observed changes would have a positive effect on life expectancy.

Based on the magnitude of the observed long-term effects on metabolic risk factors, these changes are clinically significant. For example, the 5-year decrease in SBP exceeded 3 mmHg, and a meta-analysis involving one million individuals [46] demonstrated that each 2 mmHg decrease of blood pressure is associated with a 10% reduction in stroke mortality and a 7% reduction in mortality from ischaemic heart disease (IHD) or other vascular diseases. Therefore, the blood pressure decrease in our study would, hypothetically, be associated with an approximately 15% lower risk of mortality from stroke and 10% lower mortality from IHD. In addition, other significant positive changes were observed in BMI, WC, DBP, FPG, TG, Chol, and HDL (except LDL). While, small in effect sizes, they may also be important findings because the normal clinical course for this patient group involves continued deterioration of the metabolic risk profile [47–49]. Thus, the 5-year PAP intervention period yielded both a treatment effect—with improved metabolic risk factors and attenuation of expected worsening of risk factors—and a potential preventive effect against future diseases and premature death. The improved PA level for this large patient group could potentially reduce their future healthcare needs [5,50].

The blood lipid outcome measures showed positive changes in TG, Chol, and HDL at follow-up, but, somewhat paradoxically, negative changes (increased values) in LDL. Previous research has shown that HDL is the lipid fraction most sensitive to increased PA, while PA of increased intensity is typically required to reduce LDL and TG levels [7,51,52]. However, the findings of PA-induced effects on LDL are inconsistent, and are considered to be linked to variations in human weight [51]. It has also been suggested that total LDL should be analysed according to LDL subfractions. Increased PA is reportedly followed by a decrease of atherogenic small LDL particles, in combination with an increased average size of LDL particles, which may conceal positive effects. Our present study did not include subfraction analysis of LDL, and the most commonly prescribed PA was walking at a moderate intensity level. It is possible that PA of a more vigorous intensity may have further positively affected the metabolic risk factors, particularly LDL.

In terms of HRQoL, mental health (MCS) increased while physical health (PCS) decreased over the study period. A minimal clinically important difference [53] of 3–5 points has been suggested for the SF-36 assessment [54,55]. Thus, the MCS increase of 4.2 points (p < 0.001) seems clinically relevant, while the 2.4-point decrease in PCS (p < 0.001) may not be. It is possible that PCS did not increase during the 5-year intervention due to the age distribution in the group, with a mean age of 57 years at baseline, and 26% being over 65 years of age. Among older individuals, the incidence and prevalence rates of illness and disease would normally increase during a 5-year period [4,56], possibly influencing physical function and the estimated PCS.

To our knowledge, this is the first evaluation of a PAP intervention with a 5-year follow-up period. The 48% adherence rate at 5 years obviously increased the risk of selection bias. However, similar PAP studies with shorter follow-up times have shown dropout rates of between 30–38% at 6–12 months of follow-up [57–59], and 41–48% at 2 years of follow-up [60,61]. Additionally, the large LOOK-ahead study reported very low compliance even after only 2 years of the 10-year follow-up, as indicated by the fitness values and metabolic risk factors returning to normal [24]. Swedish PAP treatment is associated with a compliance rate of around 65% at 6 months [25], which is comparable to that of regular medical interventions. Given that behavioural change is very difficult to achieve, a drop-out of about 50% at 5 years can be considered quite good. It has been suggested that the individualization of the method is important for minimizing the drop-out rate. Notably, adherence may have been influenced by logistical issues during the course of our study. At the start of the study intervention, a care choice reform [62] was implemented in the region—which clearly led to increased stress among the personnel, decreased time for working with PAP, and increased staff turnover among nurses responsible for PAP. Additionally, the earmarked financial compensation to the healthcare provider for PAP treatment was removed (but still fully funded within the general compensation), which clearly decreased PAP use at the HCC´s, during the course of the study. These changes resulted in decreased expertise in handling PAP among co-workers, and disturbed follow-up routines for the patients. Both factors probably affected the patient dropout rate.

The present findings have important clinical implications. We demonstrated that PAP has long-term effects, and these results strengthen the clinical role of PAP in healthcare. The development of major risk factor outcomes followed similar patterns over time for the three intervention groups (>150 HCC, <150 PT, and <150 HCC) and for different subgroups based on sex, age, civil status, economy, education, and smoking. Although for many outcomes, the effects waned during the last two years of intervention, we observed virtually no deterioration in the general metabolic risk profile, compared to baseline. Importantly, in previous studies, patients have emphasized that long-term increases and maintenance of PA levels require individually customized PAP treatment, with support from skilled healthcare providers [19,20,63]. At the same time, healthcare providers have requested organizational support—including more interested, clear, and supportive management; and the prioritization of more resources, particularly ear-marked time for PAP treatment [64,65]. There is presently both organizational and logistic challenges to the implementation of PAP as part of regular healthcare. Importantly, interventions promoting PA for patients in health care have been shown to be cost-effective both internationally [66–68] and in Sweden [69,70] with the possibility to save costs for the health care system. The PAP intervention used in the Gothenburg PAP-study have been considered as a low budget intervention [30]. However, no cost-effectiveness analysis of the Swedish PAP has yet been carried out, but is ongoing within the framework of the Gothenburg PAP-study. Further cost-effectiveness analyses are probably of most importance to reach a full-scale implementation of Swedish PAP.

### Strengths and limitations

One strength of the present study is that PAP treatment was conducted within the regular primary healthcare system, making the results more externally valid and applicable. Another strength, which reduced the risk of type I and type II errors, was the use of linear and quadratic mixed-effects models in the statistical analysis [71,72] estimated in latent growth curve models with repeated measure nested within individuals. All models used the FIML procedure, which includes cases with partially missing data for the study variables, and all available information was used to compute parameters.

This study also has some limitations. The selection bias was increased by the convenience sample recruitment of the study population, with patients more willing to change their PA level, and without data regarding how many patients meeting the same inclusion criteria were not included in the study. The drop-out rate of around 50% would also have affected the risk of selection bias, as discussed above. This study was based on regular daily clinical practice, in which the personnel offering PAP treatment to the patients had no extra resources, hampering consecutive inclusion in the study. Additionally, the person-centred PAP treatment method is determined by the patient’s attitude towards changing PA, and is considered most appropriate for patients in the contemplation or preparation stages [73].

## Conclusions

To our knowledge, this study is the first to evaluate the long-term (5 years) clinical effects of a PAP intervention in primary care, in physically inactive patients with metabolic risk factors. PAP was associated with positive long-term effects regarding PA level, metabolic health outcomes, and HRQoL. The long-term adherence rate was around 50% at 5 years, which is only slightly lower than in previous 2-year follow-ups, showing that lifestyle behavioural change interventions may be effective in the long-term. Despite limitations in terms of selection, the clinical implications of the findings are important, and strengthen the clinical role of PAP in healthcare. There remains a need for further research to study how to increase adherence to individualized long-term PAP treatment.

## Supporting information

S1 Table Baseline characteristics of the patients in the three subgroups. (DOCX)

S2 Table Baseline characteristics in physical activity, anthropometrics, metabolic risk factors, and health related quality of life for the patients in the three subgroups. (DOCX)

S1 File Latent growth curve model (LGCM) used separately for each of the measurement with adjustment for confounders, sex, age, civil status, economy, education, and smoking. (XLSX)

S2 File Source data file. (XLSX)
