# Supplementary material for: The effects of a 5-year physical activity on prescription (PAP) intervention in patients with metabolic risk factors
Source: PLoS One. 2022 Oct 31;17(10):e0276868. doi: 10.1371/journal.pone.0276868 (PMC9621409; doi:10.1371/journal.pone.0276868)
Supplement: S1 Table — (DOCX) [file pone.0276868.s001.docx]

| **Supporting information S1. Baseline characteristics of the patients in the three subgroups.** | | | | |
| --- | --- | --- | --- | --- |
| **Variable** | **>150 HCC**  (n=156) | **<150 PT group**  (n=98) | **<150 HCC group**  (n=92) | ***p* value** |
|  |  |  |  |  |
| **Age**^a^ **–** years | 57.8 (11.0) | 56.4 (10.2) | 57.5 (11.4) | 0.482^c^ |
| **Sex**^b^ |  |  |  | 0.078^d^ |
| Female | 92 (59.0) | 48 (49.0) | 46 (50.0) |  |
| Male | 64 (41.0) | 50 (51.0) | 46 (50.0) |  |
| **Social situation**^b^ |  |  |  | 0.298^d^ |
| Single | 52 (34.9) | 41 (43.6) | 32 (35.2) |  |
| Married/cohabit | 88 (59.1) | 49 (52.1) | 56 (61.5) |  |
| Other | 9 (6.0) | 4 (4.3) | 3 (3.3) |  |
| **Economic status**^b^ – perceived |  |  |  | 0.497^d^ |
| Good | 96 (63.2) | 56 (59.6) | 54 (59.3) |  |
| Neither nor | 41 (27.0) | 29 (30.9) | 26 (28.6) |  |
| Bad | 15 (9.9) | 9 (9.6) | 11 (12.1) |  |
| **Education**^b^ |  |  |  | 0.959^d^ |
| Elementary grade | 28 (18.3) | 14 (14.7) | 18 (20.0) |  |
| Upper secondary school | 52 (34.0) | 37 (39.0) | 30 (33.3) |  |
| University college | 71 (46.4) | 44 (46.3) | 42 (46.7) |  |
| **Tobacco**^b^ |  |  |  | 0.897^d^ |
| Smokers | 10 (6.6) | 11 (11.6) | 10 (11.0) |  |
| Non-smokers | 105 (69.1) | 59 (62.1) | 55 (60.4) |  |
| Ex-smokers | 37 (24.3) | 25 (26.3) | 26 (28.6) |  |
| **Part of metabolic syndrome**^b^ |  |  |  |  |
| Overweight/Obesity | 141 (90.4) | 87 (88.8) | 85 (92.4) | 0.964^d^ |
| Hyperglycaemia | 56 (36.1) | 35 (35.7) | 40 (44.0) | 0.500^d^ |
| Hypertension | 125 (80.1) | 79 (81.4) | 72 (78.3) | 0.957^d^ |
| Hyperlipidaemia | 83 (53.5) | 58 (59.2) | 58 (63.0) | 0.161^d^ |
| Other diagnosis |  |  |  |  |
| Mental health, depression | 24 (15.6) | 16 (16.5) | 9 (10.0) | 0.562^d^ |
| Musculoskeletal disorders | 29 (18.8) | 15 (15.5) | 11 (12.2) | 0.219^d^ |
| Other | 71 (46.1) | 40 (41.2) | 35 (38.9) | 0.266^d^ |
| **Drug treatment**^b^ |  |  |  |  |
| Overweight/Obesity | 0 (0) | 0 (0) | 1 (1.1) | 0.364^d^ |
| Hyperglycemia | 17 (11.0) | 11 (11.3) | 12 (13.3) | 0.719^d^ |
| Hypertension | 86 (55.8) | 57 (58.8) | 45 (50.0) | 0.811^d^ |
| Hyperlipidemia | 36 (23.4) | 20 (20.6) | 17 (18.9) | 0.422^d^ |
| Other drug treatment |  |  |  |  |
| Mental health, depression | 24 (15.6) | 16 (16.5) | 9 (10.0) | 0.562^d^ |
| Musculoskeletal disorders | 21 (13.6) | 15 (15.5) | 11 (12.2) | 0.943^d^ |
| Other | 61 (39.6) | 36 (37.1) | 26 (28.9) | 0.217^d^ |
|  |  |  |  |  |
| PT, physiotherapist; HCC, health care centre.  Data are given as ^a^ mean (standard deviation), as ^b^ number (percentage)  Difference between >5 p HCC group and <5 p PT/HCC group. *P*-value was determined by ^c^ an independent samples *t*-test or by ^d^ a Mann-Whitney U-test. Statistical significance was set at *p* ≤ 0.05. | | | | |
