# Supplementary material for: The effects of a 5-year physical activity on prescription (PAP) intervention in patients with metabolic risk factors
Source: PLoS One. 2022 Oct 31;17(10):e0276868. doi: 10.1371/journal.pone.0276868 (PMC9621409; doi:10.1371/journal.pone.0276868)
Supplement: S2 Table — (DOCX) [file pone.0276868.s002.docx]

| **Supporting information S2. Baseline characteristics in physical activity, anthropometrics, metabolic risk factors, and health related quality of life for the patients in the three subgroups.** | | | | |
| --- | --- | --- | --- | --- |
| **Variable** | **>150 HCC**  (n=156) | **<150 PT group**  (n=98) | **<150 HCC group**  (n=92) | ***p* value** |
|  |  |  |  |  |
| Physical activity level |  |  |  |  |
| ACSM/AHA questionnaire^a^, score | 2.0 (1.6) | 1.5 (1.6) | 1.7 (1.5) | **0.037^d^** |
| IPAQ 1-3^b^, score | 1 (1-2) | 1 (1-2) | 1 (1-2) | **0.001^e^** |
| IPAQ 1-3^c^, category |  |  |  |  |
| - Low | 66 (51.2) | 54 (69.2) | 55 (72.4) |  |
| - Moderate | 63 (48.8) | 24 (30.8) | 21 (27.6) |  |
| - High | 0 | 0 | 0 |  |
|  |  |  |  |  |
| BMI^a^, kg/m^2^ | 31.2 (4.8) | 32.5 (5.4) | 32.6 (5.5) | **0.020^d^** |
| Waist circumference^a^, cm | 106.0 (12.4) | 109.0 (14.0) | 109.9 (14.0) | **0.018^d^** |
| Blood pressure^a^, mm/Hg |  |  |  |  |
| Systolic | 136.8 (18.1) | 137.4 (18.5) | 138.2 (16.2) | 0.599^d^ |
| Diastolic | 83.4 (9.8) | 83.4 (11.1) | 81.9 (9.9) | 0.534^d^ |
| Metabolic components^a^, mmol/l |  |  |  |  |
| Fasting plasma glucose | 6.1 (1.7) | 6.3 (2.3) | 6.4 (1.8) | 0.241^d^ |
| Triglycerides | 1.6 (0.9) | 1.6 (1.0) | 1.9 (1.1) | 0.231^d^ |
| Cholesterol | 5.6 (1.4) | 5.6 (1.1) | 5.4 (1.2) | 0.555^d^ |
| HDL | 1.4 (0.4) | 1.4 (0.4) | 1.3 (0.4) | 0.356^d^ |
| LDL | 3.6 (1.2) | 3.6 (1.0) | 3.5 (1.0) | 0.565^d^ |
|  |  |  |  |  |
| HRQOL SF-36^a^, score |  |  |  |  |
| Physical component summary | 46.4 (9.8) | 44.3 (11.5) | 46.6 (7.9) | 0.375^d^ |
| Mental component summary | 45.2 (13.1) | 42.7 (13.8) | 45.2 (12.5) | 0.359^d^ |
|  |  |  |  |  |
| PT, physiotherapist; HCC, health care centre; ACSM, American College of Sports Medicine; AHA, American Heart Association; IPAQ, International Physical Activity Questionnaire; MET, metabolic equivalent; BMI, body mass index; HDL, high density lipoprotein; LDL, low density lipoprotein; HRQOL SF-36, health related quality of life 36-Item Short Form Health Survey.  Data are given as ^a^ mean (standard deviation), as ^b^ median (min-max), or as ^c^ number (percentage).  Difference between >5 p HCC group and <5 p PT/HCC group. *P*-value was determined by ^d^ an independent samples *t*-test or by ^e^ a Mann-Whitney U-test. Statistical significance was set at *p* ≤ 0.05 | | | | |
